# Retraction: Depletion of CD4^+^ CD25^+^ Regulatory T Cells Promotes CCL21-Mediated Antitumor Immunity

**DOI:** 10.1371/journal.pone.0281611

**Published:** 2023-02-02

**Authors:** The PLOS ONE Editors

Following the publication of this article [1], the authorship list was updated to include seven additional authors and to change the corresponding author [2].

The updated corresponding author (CL) recently requested retraction of [1] as they stated that this article was prepared and submitted without their knowledge or permission, and they acknowledged that some of the data published in this article [1] was also published in [3].

Following editorial assessment, additional concerns were identified, as follows:

- In Figure 5A, the anti-CD25 and CCL21+anti-CD95 panels for Ki67 appear to partially overlap despite representing different conditions.
- Tumour weight of >8g reported in Figure 2E raises concerns about welfare and the adherence of the study to internationally-accepted standards for animal research and PLOS policy.
- No humane endpoints are described despite the article reporting a survival study.

The corresponding author did not respond to requests for comment about these additional concerns by the deadline.

In light of the concerns regarding compliance with PLOS’s publication criteria, and authorship and animal research policies, the *PLOS ONE* Editors retract this article.

CL and LC agreed with the retraction. LC apologises for the issues with the published article. SZ, JQ, RL, HT, ZZ, HC, GC, YY, BL, ZS, and CZ either did not respond directly or could not be reached.

Additionally, the citation was not correctly updated during the update to the author list [2]. The correct citation is: Zhou S, Chen L, Qin J, Li R, Tao H, Zhen Z, et al. (2013) Depletion of CD4^+^ CD25^+^ Regulatory T Cells Promotes CCL21-Mediated Antitumor Immunity. PLoS ONE 8(9): e73952. https://doi.org/10.1371/journal.pone.0073952.
